# Supplementary material for: Repression of the Glucocorticoid Receptor Increases Hypoxic-Ischemic Brain Injury in the Male Neonatal Rat
Source: Int J Mol Sci. 2019 Jul 16;20(14):3493. doi: 10.3390/ijms20143493 (PMC6678481; doi:10.3390/ijms20143493)
Supplement: Supplementary file 1 [file ijms-20-03493-s001.pdf]

**Table S1**
Effect of repression of the GR on gait parameters during the CatWalk test 5, 6, and 7 weeks after hypoxic-ischemic injury

| Parameter | Paw | 5 Weeks | | | 6 Weeks | | | 7 Weeks | | |
|---|---|---|---|---|---|---|---|---|---|---|
| | | Sham | HIE + Neg. Control | HIE + siRNA GR | Sham | HIE + Neg. Control | HIE + siRNA GR | Sham | HIE + Neg. Control | HIE + siRNA GR |
| Max Intensity | RF | 131.7 ± 35.32 | 119.4 ± 44.94 | 138.3 ± 36.56 | 119.4 ± 34.64 | 114.4 ± 30.19 | 124.5 ± 33.47 | 152.8 ± 25.11 | 154.7 ± 25.03 | 165.5 ± 23.95 |
| | LF | 132.5 ± 37.61 | 115.0 ± 43.22 | 141.5 ± 36.55 | 110.9 ± 34.71 | 122.3 ± 30.07 | 124.6 ± 34.69 | 159.1 ± 26.30 | 158.2 ± 27.54 | 172.2 ± 13.05 |
| | RH | 126.0 ± 46.45 | 121.4 ± 52.28 | 132.0 ± 54.22 | 98.3 ± 36.82 | 94.42 ± 23.35 | 105.5 ± 33.34 | 126.9 ± 31.70 | 144.2 ± 31.30 | 147.5 ± 22.44 |
| | LH | 131.0 ± 46.12 | 121.9 ± 57.26 | 138.3 ± 52.22 | 88.3 ± 31.81 | 100.2 ± 22.91 | 104.5 ± 36.14 | 125.6 ± 34.87 | 143.8 ± 33.26 | 148.6 ± 24.62 |
| Max Area ($cm^2$) | RF | 0.7854 ± 0.3958 | 0.7064 ± 0.4752 | 0.8710 ± 0.3620 | 0.6514 ± 0.3012 | 0.6327 ± 0.2840 | 0.7812 ± 0.3360 | 1.0481 ± 0.3237 | 1.0401 ± 0.2787 | 1.1851 ± 0.2755 |
| | LF | 0.8152 ± 0.3614 | 0.7018 ± 0.4506 | 0.9309 ± 0.3887 | 0.6214 ± 0.2994 | 0.6949 ± 0.2502 | 0.7719 ± 0.2847 | 1.1319 ± 0.3403 | 1.1670 ± 0.2864 | 1.3014 ± 0.2465 |
| | RH | 0.5860 ± 0.3602 | 0.6099 ± 0.4582 | 0.6973 ± 0.4343 | 0.3836 ± 0.2065 | 0.4016 ± 0.1866 | 0.5127 ± 0.2553 | 0.7187 ± 0.2630 | 0.8157 ± 0.2963 | 0.9254 ± 0.2081 |
| | LH | 0.6170 ± 0.3868 | 0.6340 ± 0.4884 | 0.7637 ± 0.4456 | 0.3594 ± 0.2221 | 0.4495 ± 0.2218 | 0.5326 ± 0.2862 | 0.7069 ± 0.2862 | 0.8866 ± 0.3313 | 0.8979 ± 0.1768 |
| Print Area ($cm^2$) | RF | 0.9471 ± 0.4726 | 0.8510 ± 0.5610 | 1.0353 ± 0.4259 | 0.7571 ± 0.3392 | 0.7463 ± 0.3221 | 0.9151 ± 0.3631 | 1.2233 ± 0.3532 | 1.2348 ± 0.2900 | 1.3736 ± 0.2905 |
| | LF | 0.9841 ± 0.4336 | 0.8463 ± 0.5440 | 1.1010 ± 0.4360 | 0.7340 ± 0.3244 | 0.8259 ± 0.2838 | 0.9088 ± 0.3248 | 1.3356 ± 0.4392 | 1.3894 ± 0.3448 | 1.5510 ± 0.3647 |
| | RH | 0.7791 ± 0.5028 | 0.8155 ± 0.6320 | 0.9497 ± 0.5822 | 0.5303 ± 0.2978 | 0.5770 ± 0.2970 | 0.7233 ± 0.3529 | 0.9853 ± 0.3550 | 1.1516 ± 0.3813 | 1.2800 ± 0.2716 |
| | LH | 0.8258 ± 0.5488 | 0.8340 ± 0.6462 | 1.0061 ± 0.5872 | 0.5118 ± 0.3293 | 0.6338 ± 0.3004 | 0.7382 ± 0.3950 | 0.9690 ± 0.3886 | 1.2527 ± 0.4205 | 1.2528 ± 0.2453 |
| Print Width (cm) | RF | 1.3072 ± 0.3541 | 1.1918 ± 0.4525 | 1.3826 ± 0.2924 | 1.2774 ± 0.3811 | 1.2536 ± 0.3541 | 1.3882 ± 0.3568 | 1.7961 ± 0.2447 | 1.7384 ± 0.1590 | 1.8336 ± 0.1729 |
| | LF | 1.3160 ± 0.3697 | 1.1684 ± 0.4531 | 1.4169 ± 0.3222 | 1.2546 ± 0.3698 | 1.2995 ± 0.3294 | 1.4051 ± 0.3666 | 1.8323 ± 0.1574 | 1.8121 ± 0.1354 | 1.8499 ± 0.1269 |
| | RH | 1.2373 ± 0.4344 | 1.2405 ± 0.4909 | 1.3374 ± 0.4075 | 1.1606 ± 0.4699 | 1.2169 ± 0.4010 | 1.3773 ± 0.3975 | 1.6508 ± 0.2319 | 1.6752 ± 0.2206 | 1.7462 ± 0.1701 |
| | LH | 0.8258 ± 0.5488 | 0.8340 ± 0.6462 | 1.0061 ± 0.5872 | 0.5118 ± 0.3293 | 0.6338 ± 0.3004 | 0.7382 ± 0.3950 | 0.9690 ± 0.3886 | 1.2527 ± 0.4205 | 1.2528 ± 0.2453 |
| Print Length (cm) | RF | 1.3072 ± 0.3541 | 1.1918 ± 0.4525 | 1.3826 ± 0.2924 | 1.2774 ± 0.3811 | 1.2536 ± 0.3541 | 1.3882 ± 0.3568 | 1.7961 ± 0.2447 | 1.7384 ± 0.1590 | 1.8336 ± 0.1729 |
| | LF | 1.7350 ± 0.3022 | 1.4802 ± 0.4897 | 1.8320 ± 0.2670 | 1.6676 ± 0.3762 | 1.7577 ± 0.4293 | 1.8350 ± 0.3688 | 2.2746 ± 0.5595 | 2.3016 ± 0.4267 | 2.4043 ± 0.5717 |
| | RH | 1.2373 ± 0.4344 | 1.2405 ± 0.4909 | 1.3374 ± 0.4075 | 1.1606 ± 0.4699 | 1.2169 ± 0.4010 | 1.3773 ± 0.3975 | 1.6508 ± 0.2319 | 1.6752 ± 0.2206 | 1.7462 ± 0.1701 |
| | LH | 1.3663 ± 0.5597 | 1.3192 ± 0.5764 | 1.5019 ± 0.4860 | 1.2006 ± 0.5685 | 1.4311 ± 0.4394 | 1.4948 ± 0.5597 | 1.7518 ± 0.3629 | 2.0599 ± 0.3883 | 2.0318 ± 0.2290 |
| Stride Length (cm) | RF | 12.39 ± 0.9811 | 11.96 ± 1.3442 | 11.70 ± 1.6773 | 14.36 ± 1.3836 | 13.48 ± 1.9654 | 13.52 ± 1.4637 | 15.01 ± 1.7778 | 14.67 ± 1.6709 | 14.49 ± 0.9688 |
| | LF | 11.99 ± 1.2654 | 11.84 ± 1.3881 | 11.76 ± 1.4112 | 14.21 ± 1.5454 | 13.44 ± 1.9384 | 13.47 ± 1.3791 | 15.08 ± 1.8664 | 14.7 ± 1.3930 | 14.35 ± 0.9595 |
| | RH | 11.98 ± 1.2782 | 11.55 ± 1.4830 | 11.71 ± 2.5334 | 13.70 ± 1.8413 | 12.9 ± 2.0038 | 12.78 ± 1.7880 | 15.01 ± 1.7778 | 14.87 ± 1.5619 | 14.23 ± 1.4000 |
| | LH | 11.94 ± 1.1227 | 11.7 ± 1.0637 | 11.55 ± 1.7125 | 12.87 ± 2.1434 | 12.97 ± 1.4998 | 12.87 ± 1.9861 | 14.92 ± 1.7537 | 14.86 ± 1.4147 | 14.24 ± 0.9531 |
| Stand (s) | RF # | 0.1694 ± 0.0364 | 0.1764 ± 0.0487 | 0.1959 ± 0.0460 | 0.1602 ± 0.0361 | 0.1642 ± 0.0282 | 0.1945 ± 0.0340 | 0.1873 ± 0.0404 | 0.2011 ± 0.0330 | 0.2252 ± 0.0304 |
| | LF ## | 0.1646 ± 0.0334 | 0.1728 ± 0.0528 | 0.2040 ± 0.0531 * | 0.1523 ± 0.0316 | 0.1709 ± 0.0327 | 0.1918 ± 0.0379 * | 0.1830 ± 0.0358 | 0.2098 ± 0.0344 | 0.2266 ± 0.0248 * |
| | RH # | 0.1644 ± 0.0536 | 0.1924 ± 0.0619 | 0.2102 ± 0.0699 | 0.1516 ± 0.0507 | 0.1687 ± 0.0427 | 0.1848 ± 0.0438 | 0.1871 ± 0.0487 | 0.2178 ± 0.0458 | 0.2400 ± 0.0342 * |
| | LH # | 0.1664 ± 0.0474 | 0.1863 ± 0.0487 | 0.2098 ± 0.0614 | 0.1427 ± 0.0488 | 0.1777 ± 0.0372 | 0.1884 ± 0.0416 | 0.1925 ± 0.0490 | 0.2164 ± 0.0434 | 0.2339 ± 0.0366 |
| Swing (s) | RF | 0.1418 ± 0.0189 | 0.1563 ± 0.0301 | 0.1565 ± 0.0430 | 0.1447 ± 0.0124 | 0.1508 ± 0.0333 | 0.1498 ± 0.0219 | 0.1351 ± 0.0141 | 0.1465 ± 0.0152 | 0.1537 ± 0.0208 |
| | LF | 0.1382 ± 0.0171 | 0.1527 ± 0.0254 | 0.1522 ± 0.0420 | 0.1464 ± 0.0251 | 0.1475 ± 0.0275 | 0.1518 ± 0.0262 | 0.1409 ± 0.0170 | 0.1415 ± 0.0119 | 0.1418 ± 0.0175 |
| | RH | 0.1342 ± 0.0322 | 0.1302 ± 0.0238 | 0.1407 ± 0.0470 | 0.1356 ± 0.0316 | 0.1288 ± 0.0217 | 0.1379 ± 0.0290 | 0.1270 ± 0.0140 | 0.1234 ± 0.0107 | 0.1280 ± 0.0141 |
| | LH | 0.1295 ± 0.0223 | 0.1365 ± 0.0257 | 0.1376 ± 0.0211 | 0.1299 ± 0.0268 | 0.1261 ± 0.0181 | 0.1362 ± 0.0220 | 0.1231 ± 0.0089 | 0.1249 ± 0.0110 | 0.1275 ± 0.0116 |
| Step Cycle (s) | RF # | 0.3132 ± 0.0422 | 0.3364 ± 0.0495 | 0.3546 ± 0.0684 | 0.3083 ± 0.0355 | 0.3169 ± 0.0366 | 0.3450 ± 0.0476 | 0.3290 ± 0.0436 | 0.3528 ± 0.0409 | 0.3808 ± 0.0327 * |
| | LF ## | 0.3033 ± 0.0412 | 0.3305 ± 0.0547 | 0.3593 ± 0.0821 * | 0.3022 ± 0.0392 | 0.3213 ± 0.0347 | 0.3483 ± 0.0491 | 0.3282 ± 0.0438 | 0.3607 ± 0.0388 | 0.3752 ± 0.0381 |
| | RH # | 0.2989 ± 0.0408 | 0.3220 ± 0.0597 | 0.3540 ± 0.0724 * | 0.2842 ± 0.0398 | 0.2968 ± 0.0543 | 0.3238 ± 0.0336 | 0.3152 ± 0.0508 | 0.3285 ± 0.0301 | 0.3678 ± 0.0377 * |
| | LH ## | 0.2950 ± 0.0406 | 0.3205 ± 0.0477 | 0.3484 ± 0.0716 * | 0.2716 ± 0.0489 | 0.3039 ± 0.0490 | 0.3267 ± 0.0482 * | 0.3158 ± 0.0504 | 0.3464 ± 0.0515 | 0.3607 ± 0.0394 |
| Duty Cycle | RF | 53.77 ± 5.3495 | 52.15 ± 7.7555 | 55.82 ± 5.5822 | 52.09 ± 6.9684 | 52.45 ± 6.2253 | 55.5 ± 6.4299 | 57.93 ± 5.0189 | 56.92 ± 4.2817 | 59.04 ± 4.1547 |
| | LF # | 53.32 ± 4.4703 | 52.16 ± 7.9826 | 57.41 ± 5.2408 | 50.67 ± 6.2752 | 53.9 ± 5.9094 | 55.84 ± 5.3103 | 56.24 ± 4.1276 | 59.12 ± 4.2703 | 60.68 ± 2.6772 |
| | RH | 53.79 ± 11.8246 | 57.21 ± 8.0929 | 58.38 ± 10.3907 | 50.85 ± 12.3716 | 55.06 ± 4.9810 | 56.73 ± 7.6482 | 58.18 ± 6.1064 | 60.92 ± 4.1938 | 63.21 ± 3.0587 |
| | LH | 54.5 ± 9.7923 | 55.2 ± 7.9373 | 58.2 ± 8.3837 | 51.25 ± 10.0978 | 56.5 ± 5.2316 | 56.12 ± 7.8403 | 59.18 ± 6.0951 | 61.2 ± 5.0433 | 63.09 ± 2.6653 |
| Swing Speed (cm/s) | RF | 96.88 ± 21.7533 | 85.97 ± 22.1969 | 84.08 ± 19.1661 | 103.1 ± 15.7509 | 96.65 ± 26.4800 | 94.96 ± 16.2947 | 117.5 ± 24.1208 | 107.45 ± 13.6460 | 100.5 ± 12.7093 |
| | LF | 94.85 ± 15.3659 | 83.96 ± 18.9664 | 87.82 ± 18.4589 | 108.0 ± 23.8727 | 98.02 ± 23.3002 | 96.48 ± 15.6744 | 118.01 ± 19.7833 | 119.3 ± 41.4592 | 110.3 ± 26.2080 |
| | RH | 95.67 ± 22.0123 | 92.75 ± 24.4179 | 90.74 ± 21.4989 | 105.1 ± 18.9117 | 103.0 ± 25.7258 | 102.3 ± 20.6580 | 120.2 ± 21.1235 | 122.2 ± 15.9922 | 113.8 ± 14.2369 |
| | LH | 95.43 ± 16.7071 | 90.63 ± 22.3561 | 88.65 ± 18.9895 | 99.55 ± 26.7360 | 106.27 ± 21.8013 | 100.2 ± 26.0638 | 122.9 ± 16.4659 | 120.9 ± 15.7921 | 115.7 ± 10.5835 |

Table values are Mean ± SD, * Significant difference when compared to Sham. # Significant group difference in a 2-way ANOVA (Sham vs. Neg. Control vs. siRNA GR).
